# Shoulder elevation to improve sheath–lead coaxial alignment during transvenous lead extraction: a case report

**DOI:** 10.1093/ehjcr/ytag470

**Published:** 2026-06-17

**Authors:** Yuhei Kasai, Junji Morita, Yumetsugu Munakata, Takayuki Kitai

**Affiliations:** Department of Cardiology, Sapporo Cardiovascular Clinic, North 49, East 16, 8-1, Higashi Ward, Sapporo, Hokkaido 007-0849, Japan; Department of Cardiology, Sapporo Cardiovascular Clinic, North 49, East 16, 8-1, Higashi Ward, Sapporo, Hokkaido 007-0849, Japan; Department of Clinical Technology, Sapporo Cardiovascular Clinic, North 49, East 16, 8-1, Higashi Ward, Sapporo, Hokkaido 007-0849, Japan; Department of Cardiology, Sapporo Cardiovascular Clinic, North 49, East 16, 8-1, Higashi Ward, Sapporo, Hokkaido 007-0849, Japan

**Keywords:** Case report, Transvenous lead extraction, Tandem approach, Shoulder elevation

## Case description

**Figure 1:**
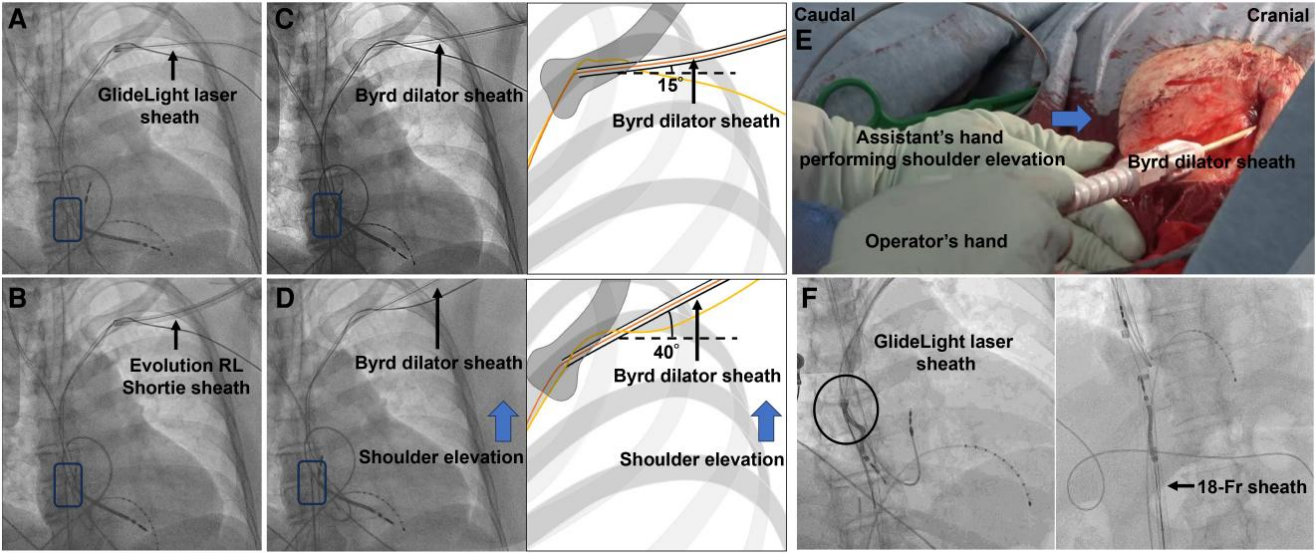
(*A*, *B*) Fluoroscopic images during dissection with a 14-Fr GlideLight laser sheath (*A*) and an 11-Fr evolution shortie RL sheath (*B*). Both images show the tandem approach in which the ventricular lead was grasped using a snare and a 0.014-inch guidewire to improve sheath–lead coaxial alignment (blue rectangle). (*C*) Fluoroscopic image (left) and schematic illustration (right) during dissection with an 11.5-Fr Byrd dilator sheath using the tandem approach (blue rectangle). However, sheath advancement remained unsuccessful. (*D*) Fluoroscopic image (left) and schematic illustration (right) during dissection with an 11.5-Fr Byrd dilator sheath. Shoulder elevation improved sheath–lead coaxiality and enabled successful sheath advancement. The inclination of the subclavian vein was increased from approximately 15° to 40° relative to the horizontal body plane. (*E*) Operative field during shoulder elevation. (*F*) Left panel: the GlideLight sheath reached the grasping site (black circle). Right panel: the detached lead tip was retrieved into the 18-Fr sheath via the right femoral vein, resulting in successful lead extraction.

A 50-year-old woman with cardiac sarcoidosis complicated by ventricular fibrillation and complete atrioventricular block had undergone implantable cardioverter defibrillator implantation (Acticor 7 DR-T DF4 ProMRI; Biotronik, Berlin, Germany) 5 years earlier. She was referred for transvenous lead extraction (TLE) and ventricular lead (Plexa ProMRI S 65; Biotronik) replacement. The TLE indication was ventricular lead dysfunction with oversensing-induced pacing inhibition in a pacing-dependent patient, resulting in recurrent presyncope.^1,2^ TLE was performed under general anaesthesia using the tandem approach (i.e. lead extraction that utilizes simultaneous superior access and femoral lead capture with a snare to optimize traction vectors) to improve coaxial alignment of the extraction sheaths.^3^ Following conventional approaches, we attempted to use both powered and non-powered sheaths, such as GlideLight laser (Philips, Andover, MA), Evolution Shortie RL, and Byrd dilator (Cook Medical, Bloomington, IN) sheaths. However, these attempts were unsuccessful (*Figure 1A, B, C*). Despite these failures, atrial lead extraction could not be performed alternatively because the atrial lead was functioning normally. We therefore attempted shoulder elevation as an alternative manoeuvre to improve sheath–lead coaxiality, which altered the lead trajectory and enabled successful Byrd sheath advancement (*Figure 1D, E*, and Supplementary material online, *Video S1*). During advancement, the lead tip detached and was retrieved into an 18-Fr sheath (Check-Flo; Cook Medical) via the right femoral vein (*Figure 1F*). A new ventricular lead and generator were successfully implanted without complications.

To our knowledge, this is the first report highlighting shoulder elevation as a simple, cost-neutral manoeuvre that improves sheath–lead coaxiality and may facilitate sheath advancement in challenging TLE procedures when conventional approaches are unsuccessful. Excessive abduction and external rotation of the shoulder should be avoided to minimize the complication risk of shoulder joint injury or dislocation. This technique may serve as a useful bailout manoeuvre for overcoming procedural impasse and facilitating successful TLE.

## Supplementary Material

ytag470_Supplementary_Data

## Data Availability

The data underlying this article are available in the article and its online Supplementary material. Further information is available from the corresponding author upon reasonable request.

## Notes

**Consent:** Written informed consent for publication of this report was obtained from the patient in accordance with the COPE guidelines.
